# Significant Difference of Immune Cell Fractions and Their Correlations With Differential Expression Genes in Parkinson’s Disease

**DOI:** 10.3389/fnagi.2021.686066

**Published:** 2021-08-17

**Authors:** Yilin Huang, Huisheng Liu, Jiaqi Hu, Chongyin Han, Zhenggang Zhong, Wei Luo, Yuhu Zhang, Fei Ling

**Affiliations:** ^1^School of Biology and Biological Engineering, South China University of Technology, Guangzhou, China; ^2^Clinical Research Institute, Foshan Hospital, Sun Yat-sen University, Foshan, China; ^3^Department of Neurology, Guangdong Neuroscience Institute, Guangdong Provincial People’s Hospital, Guangdong Academy of Medical Sciences, Guangzhou, China

**Keywords:** Parkinson’s disease, immune cells, Tregs, *PIDD1*, blood, substantia nigra

## Abstract

Parkinson’s disease (PD) is the second most neurodegenerative disease in the world. T cell infiltration in the central nervous system (CNS) has provided insights that the peripheral immune cells participate in the pathogenesis of PD. However, the association between the peripheral immune system and CNS remains to be elucidated. In this study, we analyzed incorporative substantia nigra (SN) expression data and blood expression data using the CIBERSORT to obtain the 22 immune cell fractions and then explored the molecular function to identify the potential key immune cell types and genes of PD. We observed that the proportions of naïve CD4 T cells, gamma delta T cells, resting natural killer (NK) cells, neutrophils in the blood, and regulatory T cells (Tregs) in the SN were significantly different between patients with PD and healthy controls (HCs). We identified p53-induced death domain protein 1 (*PIDD1*) as the hub gene of a PD-related module. The enrichment score of the neuron-specific gene set was significantly different between PD and HC, and genes in the neuron-related module were enriched in the biological process about mitochondria and synapses. These results suggested that the fractions of naïve CD4 T cells, gamma delta T cells, resting NK cells, and neutrophils may be used as a combined diagnostic marker in the blood, and Tregs in SN may be a potential therapeutic design target for PD.

## Introduction

Parkinson’s disease (PD) is the second most neurodegenerative disease. It is clinically defined as a progressive movement disorder, such as tremors, rigidity, and bradykinesia, and sometimes also with the presentation of non-motor symptoms like cognitive impairment (Blauwendraat et al., 2020). The pathological features of PD are the loss of dopaminergic neurons and the presence of Lewy bodies in the substantia nigra (SN). The pathogenic factors of PD are complex, and inflammation has been reported to be involved in the progress of neurodegeneration. Increasing evidence suggests that both the innate and adaptive immune systems contribute to neuron death and PD pathogenesis (Earls et al., 2019). The synucleinopathy in the central nervous system (CNS) may alter the peripheral lymphocytic profile (Earls et al., 2019). Through analyzing the immune cells in the peripheral blood and cerebrospinal fluid (CSF), researchers observed that the periphery immune microenvironment changes, which may contribute to the progress of PD (Schroder et al., 2018). Increased monocyte precursors in the blood and enriched classical monocytes but decreased activated monocytes were observed in patients with PD (Grozdanov et al., 2014). CD8 T cell fraction increases in both CSF and peripheral blood (Schroder et al., 2018), and an increased proportion of circulating CD4 T cells was observed with decreased T helper (Th) 2, Th17, and regulatory T cell (Tregs) but increased Th1 in the peripheral blood (Baba et al., 2005; Kustrimovic et al., 2018). Tregs (CD4 + Foxp3+) frequency increases in patients with AD and PD by age, accompanied by the increased inhibitory activity of Tregs (Rosenkranz et al., 2007). Effector/memory T cell (Tem) activation and Tregs dysfunction seemed to be linked to PD pathobiology and disease severity rather than disease duration (Saunders et al., 2012). Research suggests that the percentages of natural killer (NK) cells, CD3 + T cells, and CD19 + B cells, and the Treg/Th17 ratio in peripheral blood may be used to predict the risk of PD in Chinese individuals (Cen et al., 2017). In women, the Tregs/Th17 ratio in patients with PD was higher than that in normal controls, implying the neuroprotective effects of estrogen (Cen et al., 2017). However, other immune cell compositions and activities present in PD and the relationship between the periphery and CNS still require further exploration. Furthermore, it is also important to screen more reliable diagnostic biomarkers, such as the proportional difference of lymphocyte subsets.

Due to the slow progression of neurodegenerative disease pathology, the infiltration dynamic of peripheral immune cells is more difficult to quantify (Greenhalgh et al., 2020). Recently, increasing evidence suggests that the adaptive immune system, including lymphocyte subpopulations and cytokines, is involved in the PD pathogenesis (Mosley et al., 2012; Galiano-Landeira et al., 2020). Microglia are the resident myeloid cells participating in maintaining homeostasis in CNS, and activated microglia in SN were signs of PD and were involved in a persistent proinflammatory event finally leading to neuron death (Tansey and Romero-Ramos, 2019). The relative proportions of CD4+ and CD8+ subsets of αβT cells are correlated with the variation in cellular proliferation in the hippocampus (Huang et al., 2010). Tregs, a subtype of CD4 T cell, has been regarded as a protective factor during neurodegeneration in the animal models with PD by suppressing immune activation and microglia responses to α-synuclein (α-syn) aggression (Reynolds et al., 2007). A previous study suggests that the cytotoxic attack of CD8 T cells may promote and initiate neuronal death and synucleinopathy in PD (Galiano-Landeira et al., 2020). In addition, the increased activated CD8 T cell fraction was found in the CSF from patients with PD (Schroder et al., 2018). Genetic analyses indicate that the inflammatory process can be risk factor for PD. The genome-wide association studies (GWAS) have revealed the HLA region variants as the susceptible locus of PD (Hamza et al., 2010). In addition, several polymorphisms in neuroinflammation-associated genes, such as *TNF*, *IL1B*, *CD14*, and *TREM2*, are the risk of PD (Hirsch and Hunot, 2009; Rayaprolu et al., 2013). Leucine-rich repeat kinase 2 (*LRRK2*) is expressed at higher levels in the immune cells, especially in patients with late-onset PD compared to age-matched controls, which may regulate the progression of PD (Cook et al., 2017). In addition, decreased mRNA levels in CD4 + T cells of signal transducer and activator of transcription 1 (*STAT1*), driving Th1 differentiation, and nuclear receptor subfamily 4 group A member 2 (*NR4A2*)/*STAT6* involved in the Treg development were detected in patients with PD having motor complications compared to those without motor complications, which gives a new opinion of the therapeutic management of PD (Kustrimovic et al., 2018; Contaldi et al., 2020). So, further studies to elucidate the relationship between gene expression and immune cell fraction/activity, as well as to explore the common/different points between peripheral and CNS immune systems, need to be performed.

CIBERSORT (Newman et al., 2015) is an analysis tool for evaluating the proportions of immune cells from gene expression data of each sample. It has been used to analyze the immune cell infiltration in a variety of diseases, including colorectal cancer (Ge et al., 2019), lupus nephritis (Cao et al., 2019), atopic dermatitis (Felix Garza et al., 2019), and osteoarthritis (Deng et al., 2020).

The goal of the study was to characterize the immune cell fractions in SN and blood and to investigate their links. First, we accessed the immune cell fractions in the peripheral blood and the SN via CIBERSORT based on the gene expression data. Then, we further probed into the association between gene expression values and the immune cell fractions. These results suggest that the naïve CD4 T cells, gamma delta T cells, and resting NK cells, as well as neutrophils in the blood, may be a potential combined diagnostic biomarker, and Tregs in the SN may be a potential therapeutic design.

## Materials and Methods

### Data Downloaded, Processing, and Differential Gene Expression Analysis

We searched the blood microarray dataset with the keywords including “PD,” “blood,” and “Homo sapiens” from the GEO database^1^ and finally selected GSE99039, including 205 patients with idiopathic PD and 233 healthy controls (HCs). The expression matrix and platform information of GSE99039 were downloaded, and then, the expression data were annotated by gene symbols in R software. Then, we screened the differential expression genes (DEGs) between PD and HC in GEO2R with *p* < 0.05 and |log2FC| > 0.3.

We obtained the candidate microarrays that can get the unprocessed raw data (.cel file) with the keywords including “PD,” “SN,” and “Homo sapiens” from the GEO database (see text footnote 1). After preprocessing, we excluded datasets with large differences in expression values or numbers of probes. We then retained only three datasets whose expression values were approximate for the subsequence analysis. The detailed information of the datasets is in Supplementary Table 1, and the detailed meta information of each sample is in Supplementary Table 2. We downloaded the raw data and platform information of GSE20164, GSE20292, and GSE7621 and then annotated the probe id after preprocessing the raw data. Their common genes merged three expression matrices, and the batch effect between them was removed. Raw data of GSE20164, GSE20292, and GSE7621 datasets were processed through the “affy” package to read the .cel file and RMA algorithm for background correction and data normalization. Then, we normalized three gene expression matrices, and the inter batch difference was removed using the “removebatcheffect” package of a limma. The boxplots and two-dimensional PCA plots before and after removing the batch effect are shown in Supplementary Figures 3A–D. After normalization, the median expression values of these samples from three datasets were on the same level, and the PCA plot showed that the difference between them was decreased, indicating that the merged expression matrix could be used for further analysis. Then, we used the “limma” package for differential expression analysis to identify DEGs between PD and HC, with *p* < 0.05 and |log2FC| > 1.

### Functional Enrichment Analysis of DEGs

We used the gene ontology (GO) enrichment function and Kyoto Encyclopedia of Genes and Genomes (KEGG) pathway enrichment analysis. We selected the significantly enriched functional GO terms and KEGG pathways with a *p*-value < 0.05 and false discovery rate (FDR) < 0.05.

### Gene Set Enrichment Analysis and Gene Set Variation Analysis

We used the gene set enrichment analysis (GSEA) software to screen the significant KEGG pathway with a *p*-value < 0.05 and an FDR < 0.25.

The gene set variation analysis (GSVA) analysis was performed using the GSVA package with the method ssgsea. Neurons, microglia, astrocytes, and oligodendrocytes are the major cell types in CNS. Custom gene sets of the four cell types mentioned above were constructed using the top 15 high-fidelity genes from the published literature (Kelley et al., 2018). The differential analysis was performed using the limma package with the threshold adj. *p*-value < 0.05.

### Weighted Gene Coexpression Network Analysis

We constructed the coexpression networks using the weighted gene coexpression network analysis (WGCNA) package in R, with the traits including disease, age, gender, platform, datasets, and the enrichment score (ES) of the gene sets of four major CNS cell types through GSVA. First, using the pickSoftThreshold function, we selected the soft thresholding powers β-value as long as the scale-free topology fitting indices *R*^2^ reached 0.8. After obtaining the modules, we subsequently analyzed the association between modules and traits. We filtered the genes in each module with the criterion of gene significance (GS) > 0.2 and module membership (MM) > 0.8 and used a plug-in clueGO in Cytoscape software to explore the function of genes in green and cyan modules. The built-in function of the WGCNA, chooseTopHubInEachModule, is used to find the hub gene of each module. We exported the network file with the function exportNetworkToCytoscape and then visualized the network in Cytoscape with the plug-in cytoHubba.

### Statistics

Statistical analysis and visualization were performed in GraphPad Prism 8 software (version 8.0.1, GraphPad Software, United States) and R (version 3.6.3). A two-tailed Student’s *t*-test was performed to compare the difference between PD and HC groups. Correlations between two numerical variables were performed using Pearson’s correlation test. The results were considered to be statistically significant for values of *p* < 0.05.

## Results

### Result 1: Four Immune Cell Fractions in Blood From PD Compared With Those From HC Are Significantly Different and Their Correlation With DEGs in the Blood of Patients With PD

**FIGURE 1:**
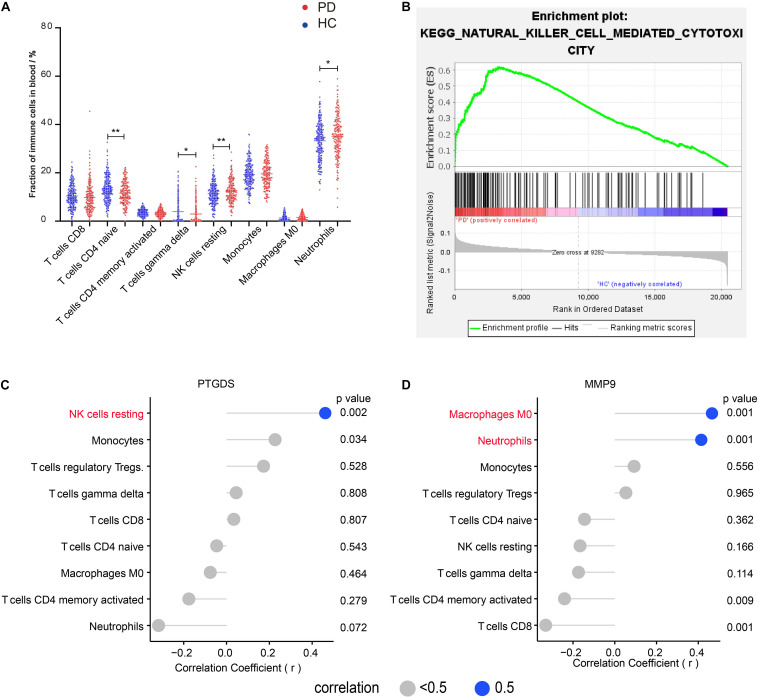
Four immune cell fractions in the blood from PD compared with those from HC are significantly different and the correlation between immune cells and DEGs in the blood of patients with PD. **(A)** The main immune cell fractions in the blood from PD and HC. The ordinate represents the immune cell fractions, and the abscissa represents the immune cell types. The blue dots represent HC samples, and the red dots represent PD samples. **(B)** KEGG pathway–NK cell-mediated cytotoxicity enriched in PD using GSEA. **(C)** Correlation between *PTGDS* and immune cells. **(D)** Correlation between *MMP9* and immune cells. *p < 0.05, **p < 0.01.

To understand the characteristics of peripheral immune cell fractions in patients with PD, we investigated the proportions of 22 immune cell types in peripheral blood using the CIBERSORT method. Compared with the immune cell proportions in blood samples of HC subjects, we found that both naïve CD4 T cells and gamma delta T cells were significantly decreased in groups with PD. In contrast, both resting NK cells and neutrophils were significantly increased (Figure 1A), which were reported separately in several previous studies by flow cytometry analysis (Niwa et al., 2012; Saunders et al., 2012; Cen et al., 2017; Zhou et al., 2020). In the blood of both PD and HC, we found that the naive CD4 + T cell fraction in a woman was significantly higher than that in a man, indicating that the naïve CD4 T cells vary between genders (Supplementary Figures 1C,D). Interestingly, GSEA of KEGG gene sets on blood expression profiles also revealed that NK cell-mediated cytotoxic pathways were enriched in PD (Figure 1B), implying that NK cells may be involved in clearing α-syn in the blood (Earls et al., 2020). To further investigate the relationship between immune cell fractions and gene expression, we screened 11 DEGs in the blood using GEO2R. We then performed the correlation analysis to confirm the correlation between DEG expression and immune cell fractions. These results showed that the mRNA level of prostaglandin D2 synthase (*PTGDS*) was positively correlated with the fraction of resting NK cells (*r* = 0.47, *p* < 0.01) (Figure 1C). In addition, the mRNA level of matrix metallopeptidase 9 (*MMP9*) was positively correlated with the fraction of neutrophils (*r* = 0.41, *p* < 0.01) and macrophages M0 (*r* = 0.47, *p* < 0.01) (Figure 1D). However, we found no correlation between any DEGs and naïve CD4 T cells and gamma delta T cells (Supplementary Figure 1B).

### Result 2: Tregs Fraction in SN Was Significantly Different Between PD and HC

**FIGURE 2:**
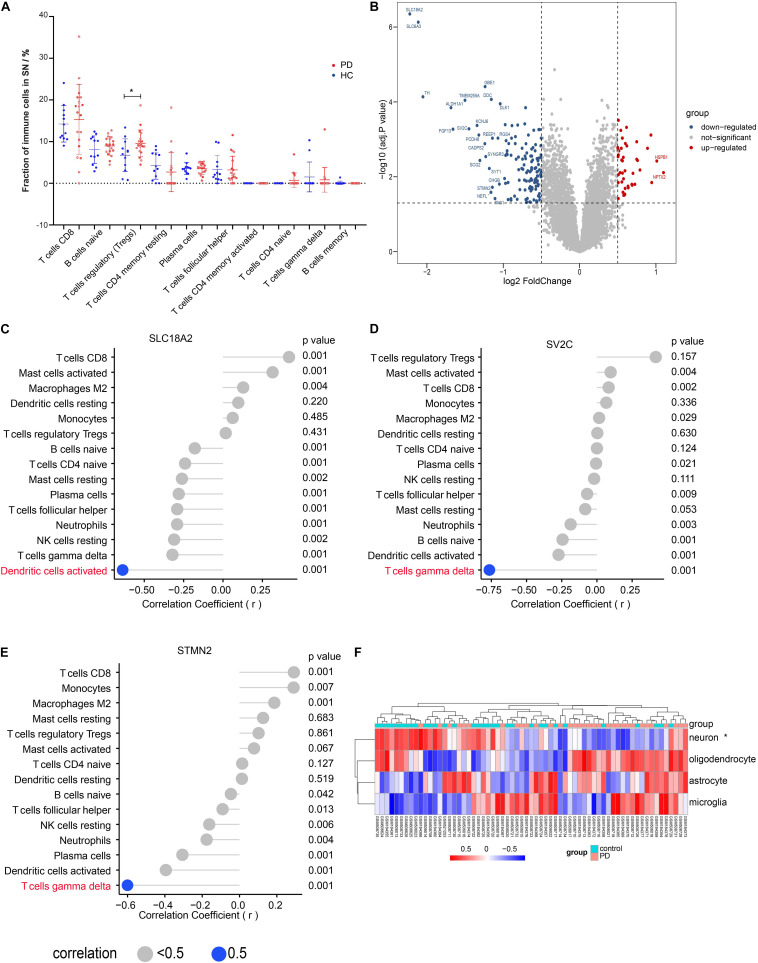
Tregs fraction in substantia nigra (SN) was significantly different between PD and HC. **(A)** The main immune cell fractions in the SN from PD and HC. The ordinate represents the immune cell fractions, and the abscissa represents the immune cell types. The blue dots represent HC samples, and the red dots represent PD samples. **(B)** Volcano plot of the differential gene analysis of SN. **(C)** Correlation between *SLC18A2* and immune cells. **(D)** Correlation between *SV2C* and immune cells. **(E)** Correlation between *STMN2* and immune cells. **(F)** The heat map of the enrichment score through the GSVA analysis. *p < 0.05.

We evaluated the peripheral immune cell fractions in SN using the CIBERSORT and found that only the fraction of Tregs was significantly different in PD compared with that in HC (Figure 2A). However, those significantly differential immune cells in the blood showed no significant difference and a low proportion in the SN (Supplementary Figure 3E). We also observed that the proportions of both macrophage M2 and CD8 T cells were higher than those of other immune cells but without statistical difference between PD and HC in the SN (Supplementary Figure 3E). Interestingly, a much more dispersion of CD8 T cell fraction values was found in the group with PD. We identified a total of 1,297 DEGs, including 702 downregulated genes and 595 upregulated genes, as shown in the volcano map (Figure 2B), among which 24 genes showed up to significant 2-fold differences in PD compared with those in HC. Aldehyde dehydrogenase family 1 (*ALDH1A1*), with reduced mRNA and protein levels in the SN of patients with PD, is associated with the progressive neurodegenerative disease (Galter et al., 2003). The polymorphism of DOPA decarboxylase (*DDC*), which can catalyze the decarboxylation of L-3,4-dihydroxyphenylalanine (DOPA) to dopamine, affects the L-DOPA response in patients with PD (Devos et al., 2014). To confirm the correlation between the immune cells and the DEGs, we performed correlation analysis on the expression values of DEGs and the immune cell fractions. Although no significant difference in the activated DC and gamma delta T cell fractions was observed (Figure 2A and Supplementary Figure 3E), correlation analysis indicated that a solute carrier family 18 member A2 (*SLC18A2*) was negatively correlated with the activated dendritic cells (DC) (*r* = −0.64, *p* < 0.01) and stathmin 2 (*STMN2*) (*r* = −0.60, *p* < 0.01), as well as synaptic vesicle glycoprotein 2C (*SV2C*) (*r* = −0.76, *p* < 0.01) was negatively correlated with gamma delta T cells, separately (Figures 2C–E), while no DEGs were identified to be correlated with Tregs (Supplementary Figure 3F). The result from the KEGG enrichment analysis showed that DEGs were enriched in PD, oxidative phosphorylation, retrograde endocannabinoid signaling, proteasome, and synaptic vesicle cycle (Supplementary Figure 4A). Biological processes enriched by DEGs included the response to hypoxia, cellular respiration, and mitochondrial respiration, and ATP synthesis coupled to electron transport (Supplementary Figure 4B). Cell composition was about the neuronal cell body and structures of mitochondrion and proteasome (Supplementary Figure 4B). To investigate the difference in the signature of major CNS cells, we performed the GSVA analysis. The results showed that the ES of the neuron-specific gene set was significantly different; however, the other three cell-specific gene sets had no statistical difference between PD and HC (Figure 2F).

### Result 3: p53-Induced Death Domain Protein 1 as a Hub Gene in the PD Module by the WGCNA Analysis in SN

**FIGURE 3:**
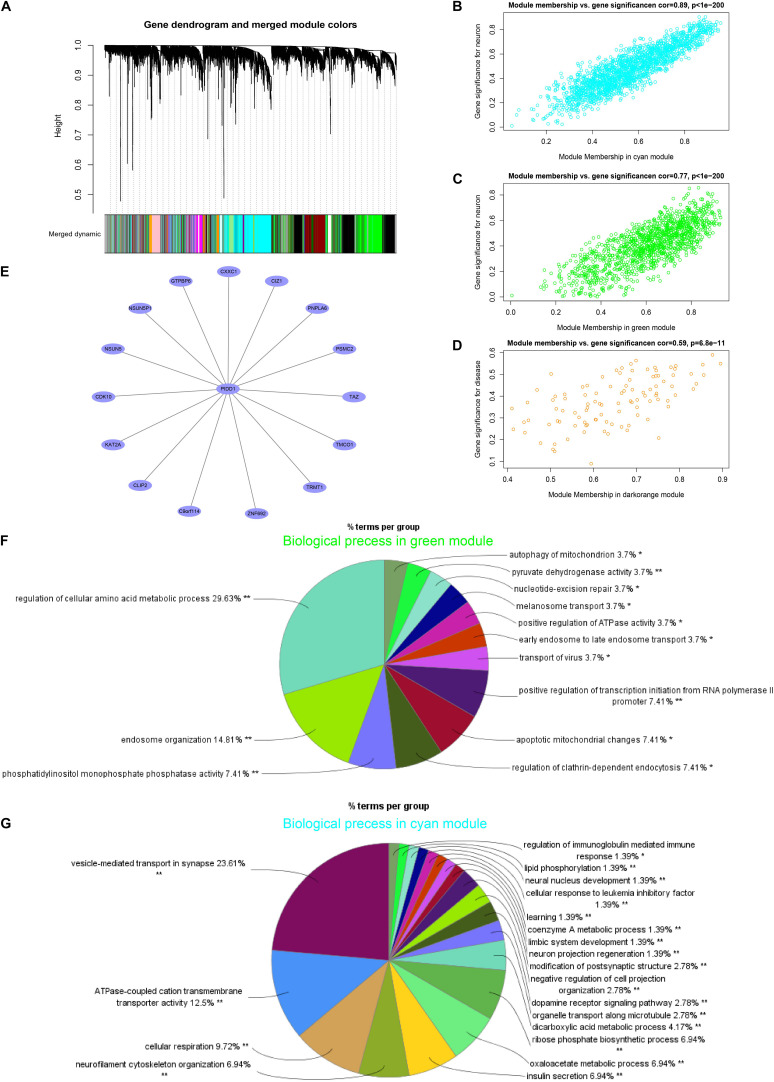
p53-induced death domain protein 1 (*PIDD1*) as a hub gene in the module of PD by the WGCNA analysis in SN. **(A)** Dendrogram of all genes clustered based on a dissimilarity measure (1-TOM). **(B–D)** Correlation between the module membership and the gene significance in cyan, green, and dark-orange modules, separately. **(E)** The network graph of the hub gene *PIDD1* of the PD-related dark-orange module. **(F,G)** The biological process of the GO enrichment analysis of filtered genes in green and cyan modules, separately. *p < 0.05, **p < 0.01.

Hierarchical clustering of gene expression data using WGCNA partitioned the data set into 28 merged modules (Figure 3A). We observed that the dark-orange module and bisque4 module were highly correlated with PD and age, separately (Supplementary Figure 5A). Both the green and cyan modules were highly correlated with the neuron-specific gene set downregulated in PD. Notably, the PD-related dark-orange module (cor = 0.59, *p* < 0.01) involved eight genes after filtering (Figure 3D), among which the p53-induced death domain protein 1 (*PIDD1*), an adaptor protein in cell death-related signaling processes, is identified as the hub gene (Figure 3E). The neuron-specific gene set is positively correlated with the green module (cor = 0.77, *p* < 0.01) and the cyan module (cor = 0.89, *p* < 0.01) (Figures 3B,C). The neuron-related green module was enriched for the biological process, mainly including the regulation of the cellular amino acid metabolic process, endosome organization, phosphatidylinositol monophosphate phosphatase activity, autophagy, and apoptotic changes of the mitochondrion, as well as melanosome transport (Figure 3F). In addition, the major biological function of the neuron-related cyan module was about the vesicle-mediated transport in synapse, ATPase-coupled cation transmembrane transporter activity, cellular respiration neurofilament cytoskeleton organization, and dopamine receptor signaling pathway, as well as neuron project regeneration (Figure 3G).

## Discussion

We characterized the immune cell fractions in the SN and blood and investigated the links between them through gaining 22 lymphocyte fractions of an individual sample in two independent cohorts of blood and SN tissues using CIBERSORT. In this study, the fractions of naïve CD4 T cells, gamma delta T cells, and resting NK cells, as well as neutrophils, were significantly different between the blood of PD and HC but not SN. However, only the Tregs fraction was different in PD SN compared with that in HC. Although we have not found a solid relationship between the peripheral and CNS immunities, we provided some views for further study.

The role of inflammation in PD is well appreciated now, obviously, and it is essential to observe for reliable and effective markers for PD. To identify the potential biomarkers of PD, we analyzed the mRNA expression array of the readily available biological samples, peripheral blood. Other studies also reported that the naïve CD4 T cells and gamma delta T cells were decreased in PD compared with those in HC (Saunders et al., 2012; Zhou et al., 2020), while NK cells and neutrophils were increased in PD (Niwa et al., 2012; Saunders et al., 2012; Cen et al., 2017), which were all observed in this study. Naïve CD4 cells can differentiate into different subtypes of Th cells, among which Th1 and Th17 are pro-inflammatory, while Th2 and Tregs are anti-inflammatory (O’Shea and Paul, 2010). Thus, the altered fraction of naïve CD4 T cells may help to understand the underlying mechanism of the neuroinflammation during PD.

The expression level of *MMP9* was positively correlated with neutrophils and macrophages M0 fraction. Matrix metalloproteinases (MMPs), mainly secreted by macrophages and neutrophils, are a family of proteolytic enzymes participating in the immune response and may facilitate the neutrophils invading the brain through the blood–brain barrier (Elkington et al., 2005; Turner and Sharp, 2016; Ritzel et al., 2018). The *PTGDS* mRNA level was positively correlated with the resting NK cells. Others found *PTGDS* to be positively correlated with the KEGG pathway NK cell-mediated cytotoxicity (Jiang et al., 2020), implying the potential relationship between *PTGDS* and NK cells during the immune response. So, these four differential distributed cells and their relationship with genes may provide new insights into investigating how the periphery immune system participates in the development of PD.

Although no remarkable difference of Tregs fractions was observed in the blood of patients with PD (Cen et al., 2017), others found CD49d + Tregs increased in PD compared to that in HC (Karaaslan et al., 2021). Tregs dysregulation and its impaired function of suppressing the activation of effector T cells were linked to the pathology of PD and disease severity (Saunders et al., 2012). In this study, the Tregs was significantly increased in SN from PD compared with that in HC. The adoptive transfer of the activated Treg attenuated astrogliosis and microglia inflammation with concomitant neuroprotection, together with the upregulation of the brain-derived neurotrophic factor and the glial cell-derived neurotrophic factor expression and the downregulation of proinflammatory cytokines and oxidative stress (Liu et al., 2009). So, Tregs was thought to be neuroprotective. Thus, the Tregs fraction that is increased in PD may not be a trigger factor of the pathology of PD but a compensation mechanism to avoid the hyperactive inflammatory response under the pathological state. In these results, other T cell fractions had no statistical difference in SN between PD and HC, but we found that the frequency value of CD8 T cells was more dispersed in PD. CD4 and CD8 T cells had been found in the aged control SN, with only the density but not the percentage of CD8 T cells increasing in PD. They proposed that CD8 T cells contribute to the nigral dopaminergic neuron dysfunction and death in PD before apparent Lewy bodies appear (Galiano-Landeira et al., 2020). Although NK cells had been considered a highly relevant cell type in PD because of the involvement in clearing α-syn in the mouse model (Earls and Lee, 2020; Earls et al., 2020), few NK cells were observed in PD and HC SN. No significant differences in the activated DC and gamma delta T cell fractions were observed. However, we found that *SLC18A2* was negatively correlated with DC, and *STMN2* and SV2C were negatively correlated with gamma delta T cells. Decreased *SLC18A2*, *STMN2*, and *SV2C* expressions were found in the brain tissues from patients with PD. All three genes were considered a potential contributor to the pathogenesis of PD by regulating the synaptic vesicle function and then dopamine neuron function (Dunn et al., 2017; Lohr et al., 2017; Cho et al., 2019; Wang et al., 2019). The activated DC and gamma delta T cells may also have some potential links with the pathology of PD, which remains to be further explored.

Furthermore, we also analyzed the molecular function and coexpression network in SN from PD. These GSVA results showed that the set neuron-specific genes ES were decreased in PD compared with those in HC, indicating that mussy pathways altered may be caused by the neuron loss. *PIDD1*, as the hub gene of PD-related module, was slightly upregulated in PD and was a component of the DNA damage/stress response pathway that functions downstream of p53/*TP53* and could either promote cell survival or apoptosis (Tinel and Tschopp, 2004; Janssens et al., 2005; Tinel et al., 2006). Nuclear factor kappa-light-chain-enhancer of activated B cells (NF-κB) is a prototypical proinflammatory signaling molecule (Dolgacheva et al., 2019). *PIDD1* may promote the inflammatory process by activating the NF-κB signaling pathway (Zhong et al., 2017). Another two genes in the PD-related module, proteasome 26S subunit-ATPase 2 (*PSMC2*) and lysine acetyltransferase 2A (*KAT2A*), may also play roles in the neurodegenerative progression. *PSMC2*, involved in protein homeostasis, was downregulated in PD compared to that in HC in this study. Another study found that the *PSMC2* protein level decreased with age in neural stem cells (NSCs) derived from the subventricular zone on postnatal 7th day, 1 month, and 12-month-old mice suggested that *PSMC2* might accelerate aging or progression of PD (Wang et al., 2016). *KAT2A* was an essential regulator of the hippocampal memory consolidation by regulating a highly interconnected hippocampal gene expression network linked to neuroactive receptor signaling via the NF-κB pathway (Stilling et al., 2014) and played a role in regulating T cell activation (Gao et al., 2017). *PIDD1* as the hub gene may also take part in the progression of PD via regulating *PSMC2* or *KAT2A*. However, how these genes affect the progression of PD is undefined and remains to be further explored. The function of genes in the neuron-related modules suggested that these biological processes change, especially those regarding mitochondrion and synaptic functions, which contribute to neuron death and neurodegeneration in PD.

However, the study has some limitations. First, the results may be more reliable if we enlarged the sample size. Second, the blood and SN samples are not from the same individual, which affects our research in finding a similarity between blood and SN. Third, the reference signature matrix LM22 had only 22 lymphocyte subsets, and we may find links between blood and SN if we have more detailed lymphocyte subsets. Finally, further study to clarify the relationship between genes and immune cells and their underlying effect on the development of PD needs to be conducted in animal models and *in vitro* cell experiments.

## Conclusion

In conclusion, we identified that the proportions of naïve CD4 T cells, gamma delta T cells, resting NK cells, and neutrophils were significantly different in the blood of PD compared with those of HC. Only Tregs fraction was observed to be significantly increased in PD SN compared to that in HC, and we identified *PIDD1* as a hub gene correlated with PD. Thus, the difference in the lymphocyte subsets in the blood between PD and HC may be used as combined diagnostic biomarkers, and Tregs in SN may be a potential therapeutic target of PD.

## Data Availability Statement

Publicly available datasets were analyzed in this study. This data can be found here: https://www.ncbi.nlm.nih.gov/geo/query/acc. cgi?acc=GSE99039; https://www.ncbi.nlm.nih.gov/geo/query/acc.cgi?acc=GSE20164; https://www.ncbi.nlm.nih.gov/geo/query/acc.cgi?acc=GSE20292; and https://www.ncbi.nlm.nih.gov/geo/query/acc.cgi?acc=GSE7621.

## Author Contributions

All authors listed have made a substantial, direct and intellectual contribution to the work, and approved it for publication.

## Conflict of Interest

The authors declare that the research was conducted in the absence of any commercial or financial relationships that could be construed as a potential conflict of interest.

## Publisher’s Note

All claims expressed in this article are solely those of the authors and do not necessarily represent those of their affiliated organizations, or those of the publisher, the editors and the reviewers. Any product that may be evaluated in this article, or claim that may be made by its manufacturer, is not guaranteed or endorsed by the publisher.

## Footnotes

1
https://www.ncbi.nlm.nih.gov/geo/
