# Supplementary material for: Significant Difference of Immune Cell Fractions and Their Correlations With Differential Expression Genes in Parkinson’s Disease
Source: Front Aging Neurosci. 2021 Aug 17;13:686066. doi: 10.3389/fnagi.2021.686066 (PMC8416258; doi:10.3389/fnagi.2021.686066)
Supplement: Supplementary file 1 [file Data_Sheet_1.pdf]

Supplementary Material

1 Supplementary Figures and Tables

1.1 Supplementary Figure lengends

Supplementary Figure1. (A) The other fourteen immune cell fractions in the blood from PD and HC. The ordinate represents the immune cell fractions, and the abscissa represents the immune cell types. The blue dots represent HC samples, and the red dots represent PD samples. (B) Correlation between DEGs and immune cells in the blood. (C, D) Some immune cell fractions between genders in the blood from PD and HC samples, separately. The green dots represent female samples, and the purple dots represent male samples. *p < 0.05, **p < 0.01, ***p < 0.001.

Supplementary Figure 2. KEGG pathways enriched in PD using GSEA.

Supplementary Figure 3. (A, B) Boxplot of samples in GSE20164, GSE20292, GSE7621 before and after removing batch effect. (C, D) Principal Component Analysis (PCA) plot of GSE20164, GSE20292, GSE7621 before and after removing batch effect. (E) The other twelve immune cell fractions in the substantia nigra from PD and HC. The ordinate represents the immune cell fractions, and the abscissa represents the immune cell types. The blue dots represent HC samples, and the red dots represent PD samples. (F) Correlation between DEGs and immune cells in the substantia nigra. *p < 0.05, **p < 0.01, ***p < 0.001.

Supplementary Figure 4. (A) KEGG pathways enriched by the DEGs in substantia nigra. (B) GO terms enriched by the DEGs in the substantia nigra.

Supplementary Figure 5. (A) Analysis of the scale-free fit index (left) and the mean connectivity (right) for various soft-thresholding powers. (B) The *PIDD1* mRNA expression level in PD and HC substantial nigra samples. (C) The heatmap of the relationship between modules and traits. The abscissa is the mRNA level. HC, 32 healthy control substantial nigra samples; PD, 33 substantial nigra samples from patients with Parkinson's disease. *p < 0.05, **p < 0.01, ***p < 0.001.

Supplementary Figure 6. (A-O) The mRNA level of *PIDD1* connected genes in PD and HC substantial nigra samples. HC, 32 healthy control substantial nigra samples; PD, 33 substantial nigra samples from patients with Parkinson's disease. *p < 0.05, **p < 0.01, ***p < 0.001.

1.2 Supplementary tables

Supplementary Table1. Gene Expression Omnibus expression datasets using in the study of Parkinson' disease

GEO Accession	Number of PD patients (PD)	Number of Healthy controls (HC)	Platform	Tissue
GSE20164	6	5	GPL96 [HG-U133A] Affymetrix Human Genome U133A Array	Substantial nigra
GSE20292	11	18	GPL96 [HG-U133A] Affymetrix Human Genome U133A Array	Substantial nigra
GSE7621	16	9	GPL570 [HG-U133_Plus_2] Affymetrix Human Genome U133 Plus 2.0 Array	Substantial nigra
Total	33	32		
GSE99039	205	233	GPL570 [HG-U133_Plus_2] Affymetrix Human Genome U133 Plus 2.0 Array	Peripheral blood

Supplementary Table 2. Substantial nigra sample's informations from GSE20164, GSE20292, GSE7621.

Sample accession	disease state	Tissue	Age	Gender	Platform	GEO accession
GSM508708	control	substantia nigra	57	M	GPL96	GSE20292
GSM508710	PD	substantia nigra	73	M	GPL96	GSE20292
GSM508711	PD	substantia nigra	80	F	GPL96	GSE20292
GSM508712	PD	substantia nigra	84	F	GPL96	GSE20292
GSM508713	PD	substantia nigra	70	F	GPL96	GSE20292
GSM508714	PD	substantia nigra	82	F	GPL96	GSE20292
GSM508715	PD	substantia nigra	70	M	GPL96	GSE20292
GSM508716	PD	substantia nigra	80	M	GPL96	GSE20292
GSM508717	control	substantia nigra	94	F	GPL96	GSE20292
GSM508718	PD	substantia nigra	70	M	GPL96	GSE20292
GSM508720	control	substantia nigra	79	M	GPL96	GSE20292
GSM508721	control	substantia nigra	67	M	GPL96	GSE20292
GSM508722	control	substantia nigra	54	M	GPL96	GSE20292
GSM508723	control	substantia nigra	73	F	GPL96	GSE20292
GSM508724	control	substantia nigra	82	M	GPL96	GSE20292
GSM508725	control	substantia nigra	72	F	GPL96	GSE20292
GSM508726	control	substantia nigra	73	F	GPL96	GSE20292
GSM508728	PD	substantia nigra	75	M	GPL96	GSE20292
GSM508729	control	substantia nigra	74	M	GPL96	GSE20292
GSM508730	control	substantia nigra	72	F	GPL96	GSE20292
GSM508731	PD	substantia nigra	79	F	GPL96	GSE20292
GSM508732	PD	substantia nigra	67	M	GPL96	GSE20292
GSM508733	control	substantia nigra	75	M	GPL96	GSE20292
GSM508734	control	substantia nigra	81	M	GPL96	GSE20292
GSM508735	control	substantia nigra	55	M	GPL96	GSE20292
GSM521253	control	substantia nigra	59	M	GPL96	GSE20292
GSM606624	control	substantia nigra	41	M	GPL96	GSE20292
GSM606625	control	substantia nigra	42	M	GPL96	GSE20292
GSM606626	control	substantia nigra	53	M	GPL96	GSE20292
GSM506013	control	substantia nigra	72.4	F	GPL96	GSE20164
GSM506014	control	substantia nigra	88	F	GPL96	GSE20164
GSM506015	PD	substantia nigra	74	NA	GPL96	GSE20164
GSM506016	PD	substantia nigra	83	NA	GPL96	GSE20164
GSM506017	PD	substantia nigra	83.6	NA	GPL96	GSE20164
GSM506018	PD	substantia nigra	79	NA	GPL96	GSE20164
GSM506019	control	substantia nigra	72	F	GPL96	GSE20164
GSM506020	control	substantia nigra	80.7	F	GPL96	GSE20164
GSM506021	PD	substantia nigra	87	NA	GPL96	GSE20164
GSM506022	PD	substantia nigra	82.4	NA	GPL96	GSE20164
GSM506023	control	substantia nigra	90	M	GPL96	GSE20164

Supplementary Table 2. (continued)

GSM184354	control	substantia nigra	NA	F	GPL570	GSE7621
GSM184355	control	substantia nigra	NA	M	GPL570	GSE7621
GSM184356	control	substantia nigra	NA	F	GPL570	GSE7621
GSM184357	control	substantia nigra	NA	F	GPL570	GSE7621
GSM184358	control	substantia nigra	NA	F	GPL570	GSE7621
GSM184359	control	substantia nigra	NA	F	GPL570	GSE7621
GSM184360	control	substantia nigra	NA	M	GPL570	GSE7621
GSM184361	control	substantia nigra	NA	M	GPL570	GSE7621
GSM184362	control	substantia nigra	NA	M	GPL570	GSE7621
GSM184363	PD	substantia nigra	NA	M	GPL570	GSE7621
GSM184364	PD	substantia nigra	NA	M	GPL570	GSE7621
GSM184365	PD	substantia nigra	NA	M	GPL570	GSE7621
GSM184366	PD	substantia nigra	NA	M	GPL570	GSE7621
GSM184367	PD	substantia nigra	NA	M	GPL570	GSE7621
GSM184368	PD	substantia nigra	NA	F	GPL570	GSE7621
GSM184369	PD	substantia nigra	NA	M	GPL570	GSE7621
GSM184370	PD	substantia nigra	NA	M	GPL570	GSE7621
GSM184371	PD	substantia nigra	NA	F	GPL570	GSE7621
GSM184372	PD	substantia nigra	NA	M	GPL570	GSE7621
GSM184373	PD	substantia nigra	NA	M	GPL570	GSE7621
GSM184374	PD	substantia nigra	NA	F	GPL570	GSE7621
GSM184375	PD	substantia nigra	NA	M	GPL570	GSE7621
GSM184376	PD	substantia nigra	NA	M	GPL570	GSE7621
GSM184377	PD	substantia nigra	NA	M	GPL570	GSE7621
GSM184378	PD	substantia nigra	NA	M	GPL570	GSE7621

Supplementary Table 3. Four immune cell fractions in the blood

Cell type	HC (mean of immune cell fraction) / %	PD (mean of immune cell fraction) / %	P-value
naive CD4 T cells	13.06	11.57	0.0017
gamma delta T cells	3.917	2.924	0.0270
resting NK cells	11.34	12.56	0.0049
Neutrophils	33.23	35.07	0.0188
