# Supplementary material for: Significant Difference of Immune Cell Fractions and Their Correlations With Differential Expression Genes in Parkinson’s Disease
Source: Front Aging Neurosci. 2021 Aug 17;13:686066. doi: 10.3389/fnagi.2021.686066 (PMC8416258; doi:10.3389/fnagi.2021.686066)

Supplementary Figure 1

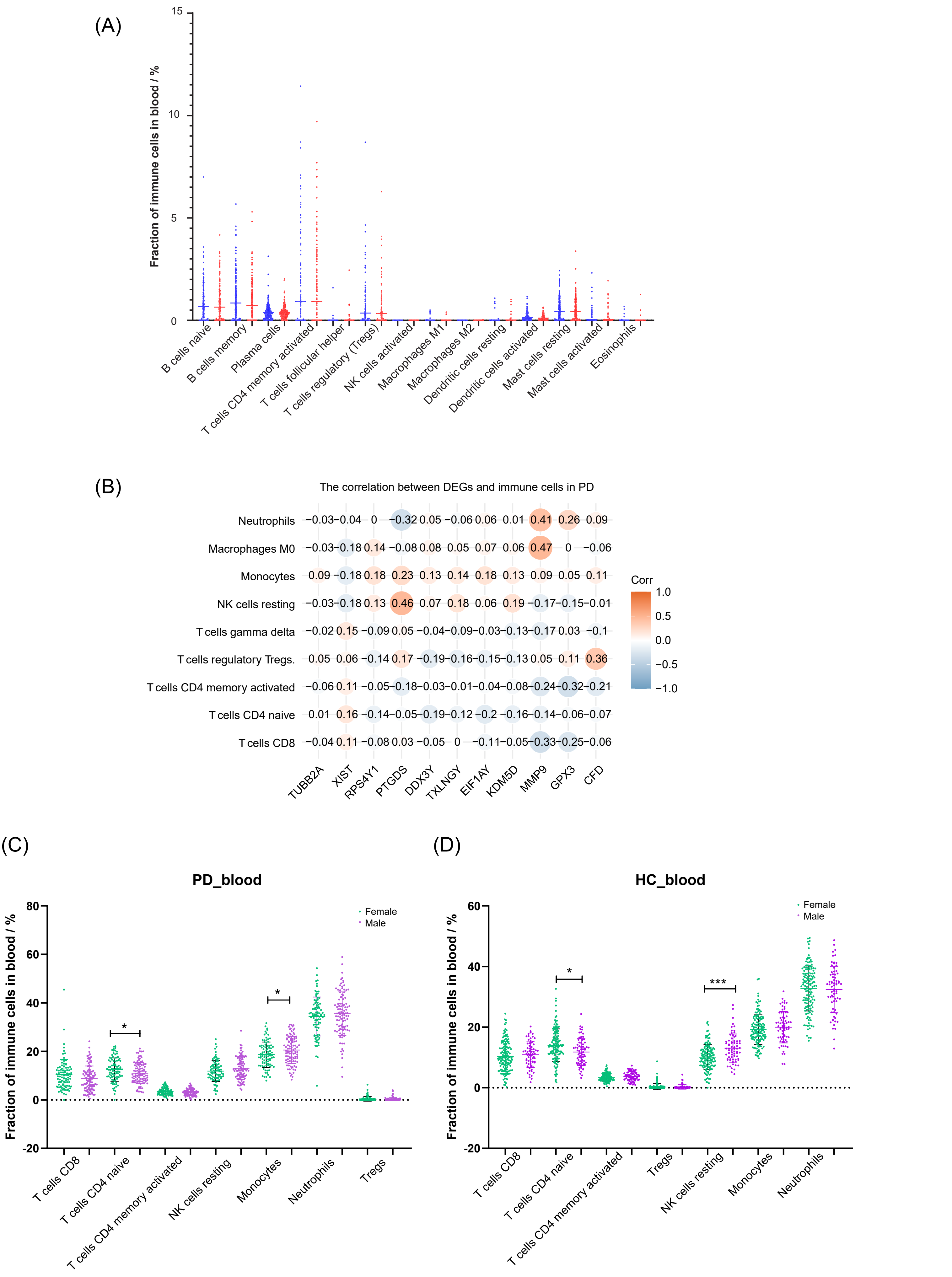

Supplementary Figure 2

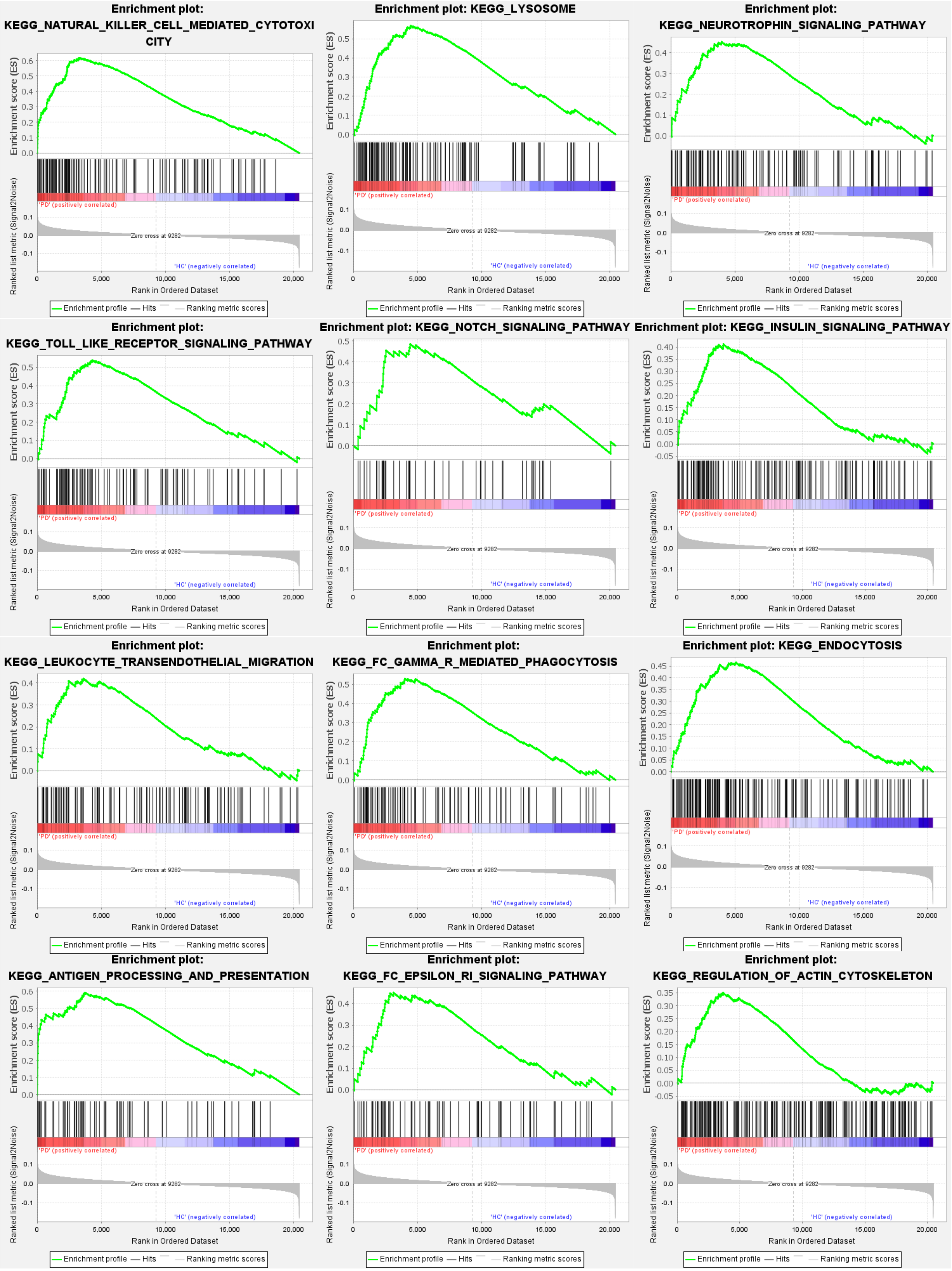

Supplementary Figure 3

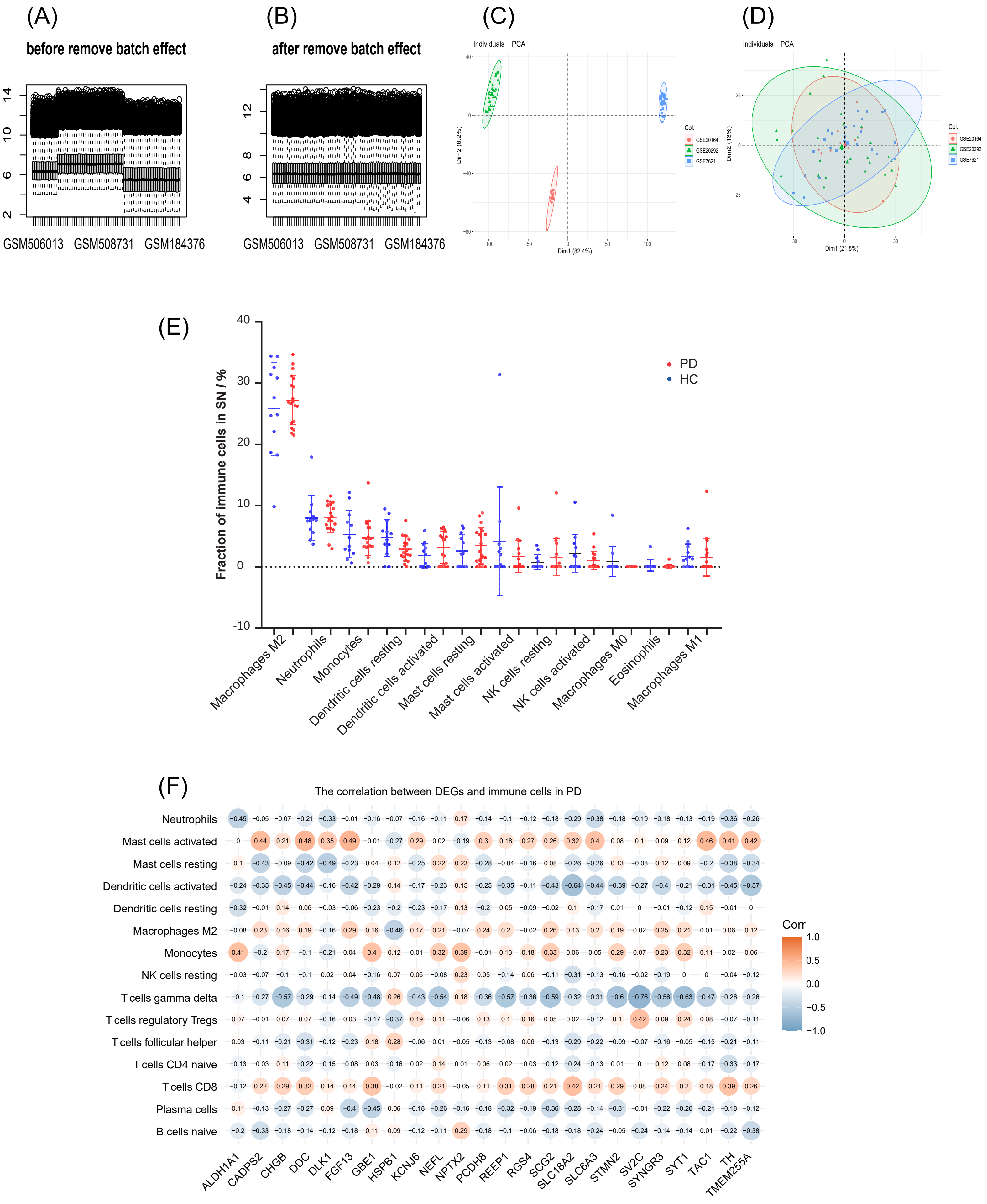

Supplementary Figure 4

(A)

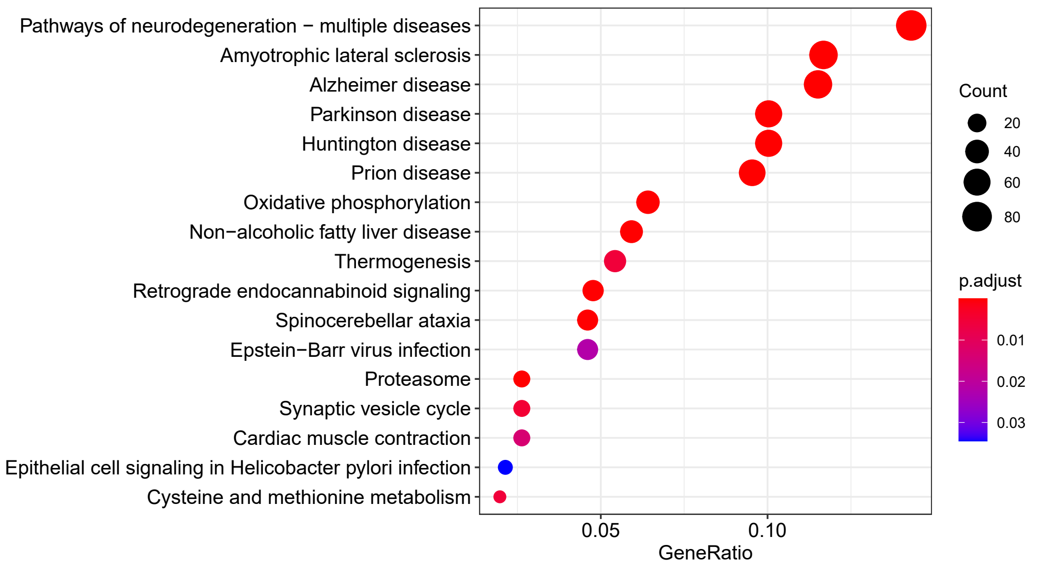

(B)

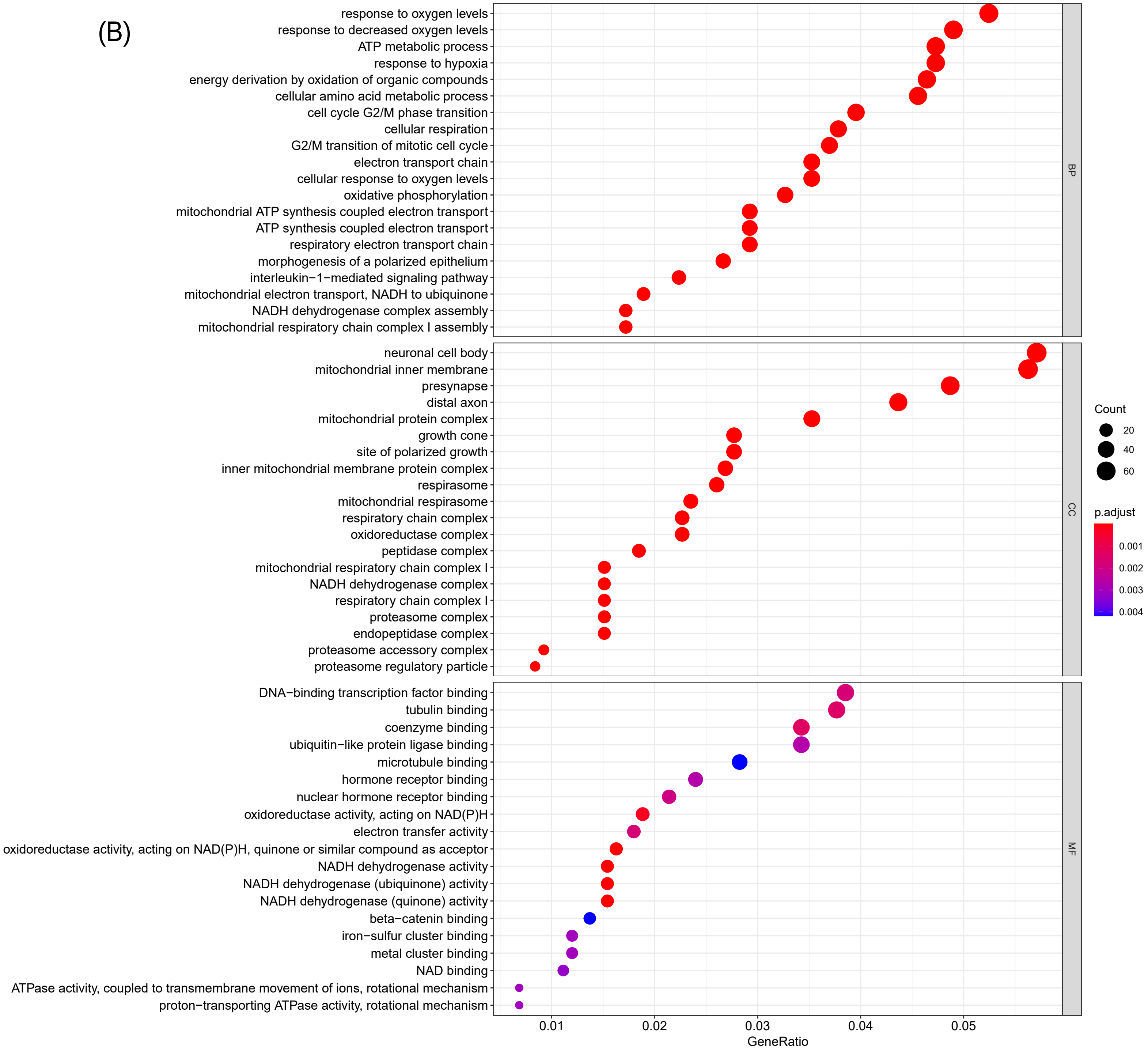

Supplementary Figure 5

(A)

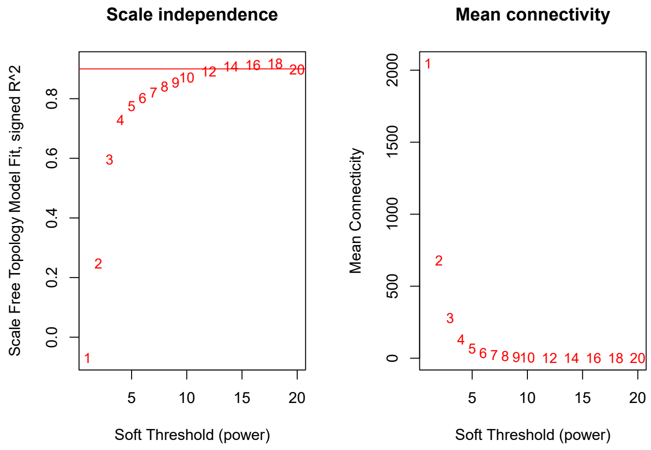

(B)

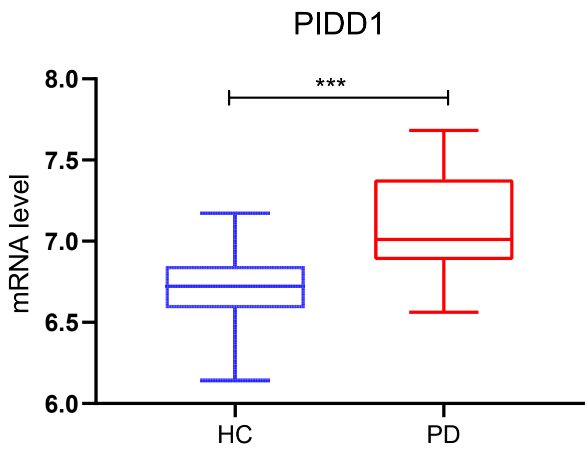

(C)

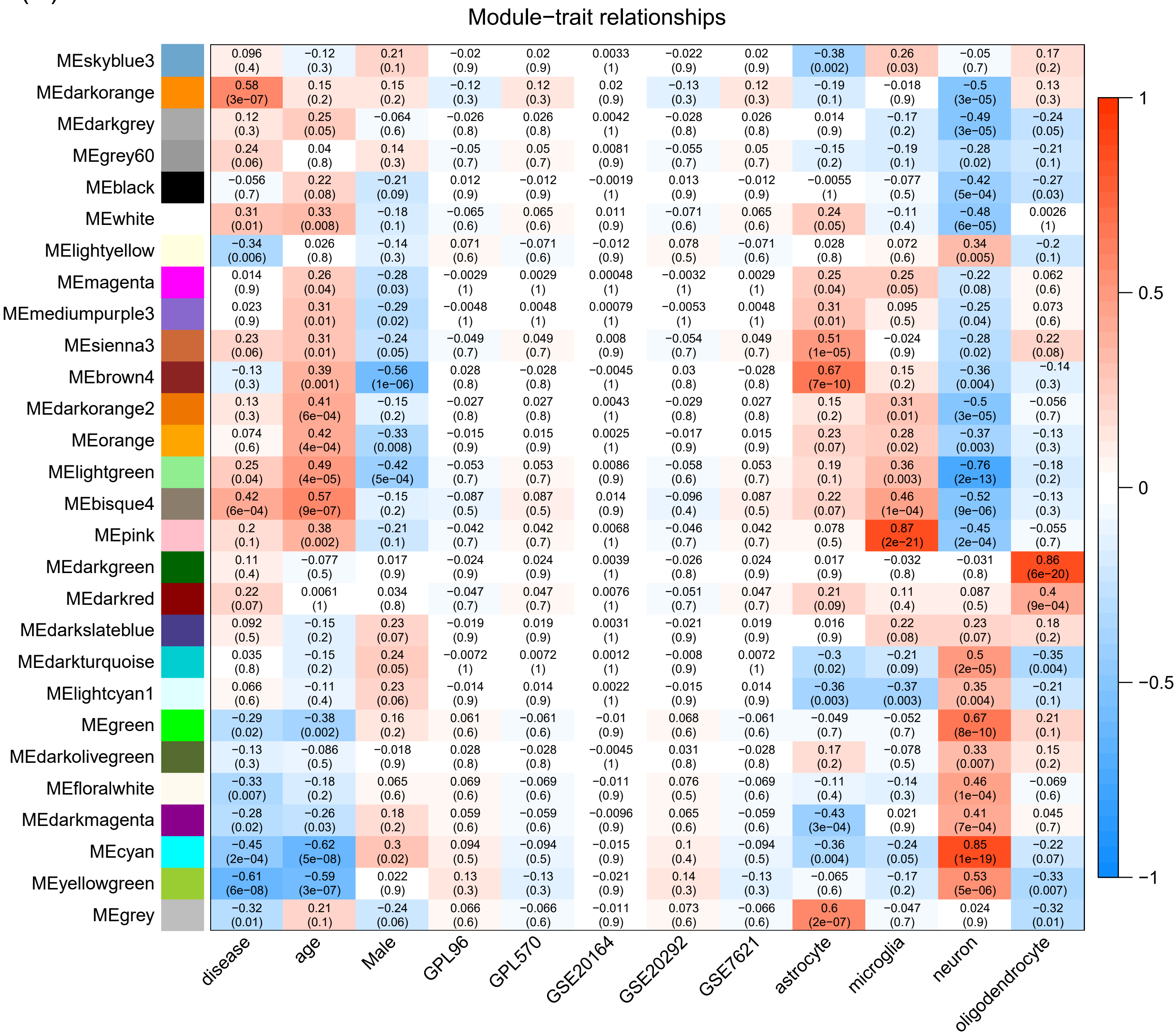

Supplementary Figure 6

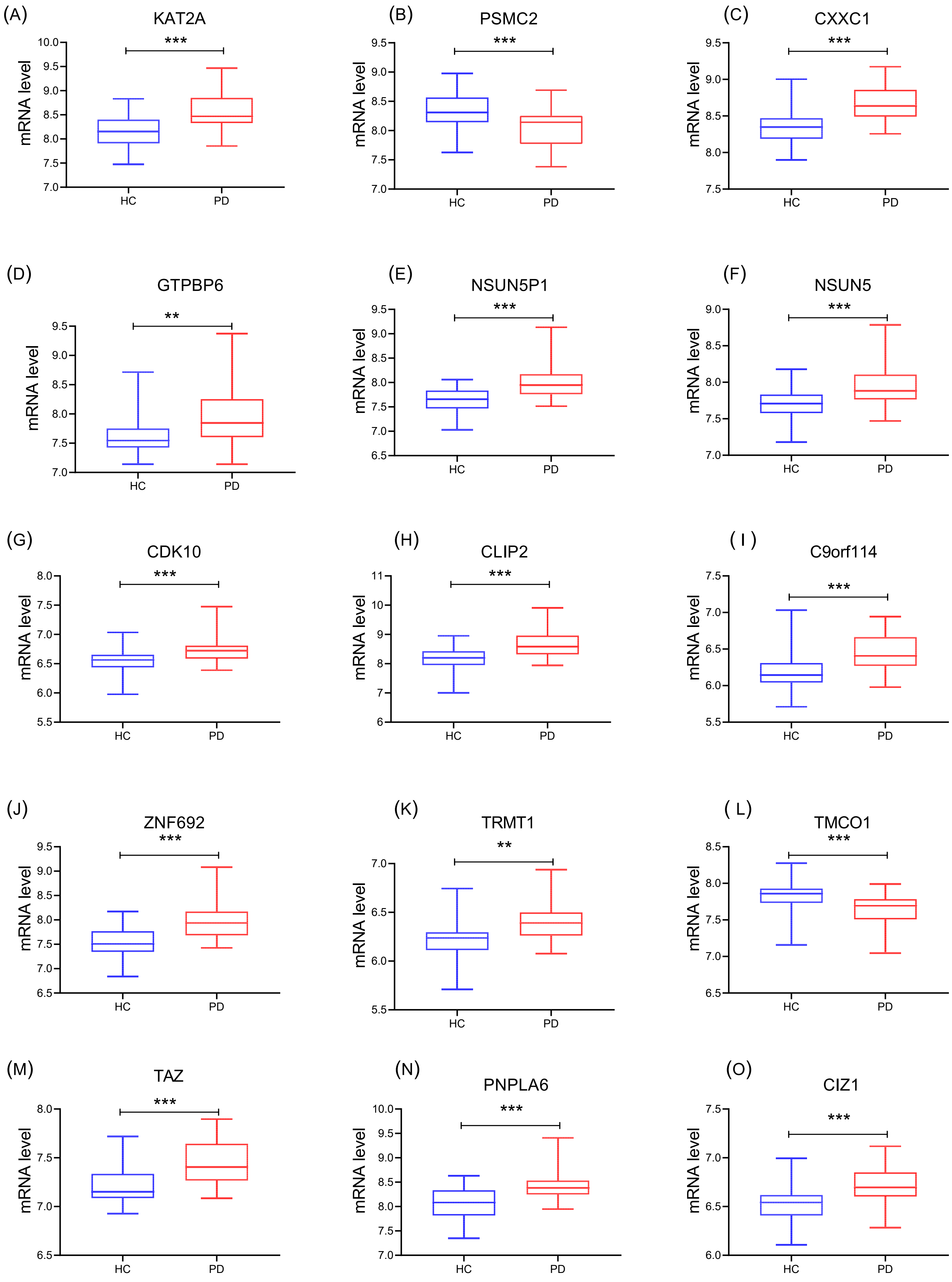

Supplement: Supplementary file 2 [file Data_Sheet_2.PDF]
